# Changes in movement organization and control strategies when learning a biomechanically constrained gait pattern, racewalking: a PCA study

**DOI:** 10.1007/s00221-016-4853-8

**Published:** 2016-12-10

**Authors:** L. Majed, A. M. Heugas, I. A. Siegler

**Affiliations:** 1 Sport Science Program, College of Arts and Sciences, Qatar University, P.O. Box 2713, Doha, Qatar; 2 CIAMS, Univ. Paris-Sud, Université Paris-Saclay, 91405 Orsay Cedex, France; 3 CIAMS, Université d’Orléans, 45067 Orléans, France

**Keywords:** Motor learning, Gait analysis, Racewalking, Movement reorganization, PCA, Control dimension

## Abstract

Combining advances from gait analysis and motor learning fields, this study aims to examine invariant characteristics and practice-related changes in the control of walking gait when learning a biomechanically constrained pattern, racewalking (RW). RW’s regulation imposes a straightened knee at the stance phase which differentiates it qualitatively from normal walking. Using 3D motion analysis, we computed key kinematic variables from a whole-body model. Principal component analysis was then used as a tool to evaluate the evolution of normal walking synergies (S0) immediately at the first practice session (S1) and further with practice (S1–S4). Before the start of practice, normal walking was characterized by two predominant control dimensions explaining an upper-extremities/antero-posterior component (PC1) and a lower-extremities/vertical component (PC2). Compared to normal walking, the immediate increase at S1 in the number of PCs needed to explain a significant portion of movement variance could be suggestive of a recruitment of a task-specific component. With practice, the significant decrease in the variance accounted for by PC1 and in the correlations between many variables could indicate a destabilization of spontaneous tendencies to facilitate the adoption of more task-specific coordinative pattern. PC2 seemed to be reinforced with practice where a significant increase in its explained variance was observed. In sum, this study shows that common features in the gait control are preserved with practice, and the movement reorganization, however, seems rather defined by shifts in the relative contribution of some variables within each PC.

## Introduction

The apparent simplicity of producing whole-body actions, like walking for instance, hides underlying complex processes in which the many redundant degrees of freedom (DoF) need to be coordinated and controlled (Bernstein 1967; Newell 1986). In the field of motor control and learning, understanding the strategies used by the central nervous system (CNS) to solve the complexity problem has become a central issue (Newell and Vaillancourt 2001). Several studies have examined the existence of invariant principles in the organization of *mechanical* DoF by studying kinematic properties of body joints and segments’ spatial configurations (Vereijken et al. 1992; Temprado et al. 1997; Caillou et al. 2002; Majed et al. 2012). Among widely examined principles, the “freezing-releasing” strategy (Bernstein 1967) suggests an initial “freezing” of the DoF, understood as rigid couplings between the latter to reduce the complexity problem. Progression in skill is then associated with a release of the constraints imposed early in practice (i.e., freezing) thus organizing the DoF into coordinated action. Although considerable advances have been made in that field, taken together, studies failed to generalize on invariant features of movement control with skill acquisition given the importance of task-specific and environmental constraints (Newell and Vaillancourt 2001; Ko et al. 2003; Majed et al. 2012). Other studies in motor learning have focused on the *dynamical* properties of the DoF to examine the synergies resulting from the organization of *mechanical* DoF (Mitra et al. 1998; Hong and Newell 2006). This was referred to as *dynamical* DoF (Mitra et al. 1998) that reflects the dimension of control or in other terms “coordinative structures” or “units of coordination” (Daffertshofer et al. 2004; Torres-Oveido and Ting 2010).

The use of principal component analysis (PCA), a linear multivariate statistical method based on correlation analysis, has proven to be effective in reducing the redundancy of large kinematic datasets and extracting relevant hidden structures and regularities in the movement variance (Daffertshofer et al. 2004; Rein et al. 2010). For instance, PCA was successfully used to determine the number of independent control dimensions (i.e., principal component, PC) encompassing the motion of several body parts that are believed to be controlled as a single unit. Many studies were able to identify different skill levels using PCA, for example in racewalkers, cello players and pianists (Dona et al. 2009; Verrel et al. 2013; Winges and Furuya 2015). Other studies have focused on changes in control strategies with the learning process where a “recruitment-suppression” principle has been formulated and tested by researchers (Newell and van Emmerik 1989; Haken 1996; Chen et al. 2005; Verrel et al. 2013). According to the theory, beginners recruit additional movement dimension(s) to control the production of a desired complex action and as the level of practice increases, the suppression of control dimension(s) would reflect the coupling of certain DoF into single units of action. However, this strategy has also failed to generalize to the learning of different motor skills (Caillou et al. 2002; Hong and Newell 2006). Hong and Newell (2006) reported no changes in the number of relevant PCs with practice on a ski simulator; however, they observed a reorganization in the relative contribution of the movement variables within each PC. Similarly, in a recent study examining changes with practice in hand movement patterns on a digital piano, Furuya et al. (2014) showed that among the two main PCs (i.e., retained PCs), only the second one was sensitive to practice while the first was practice independent.

In the present study, we challenged to examine changes in control strategies using PCA when learning a constrained walking gait pattern, namely racewalking. Compared to other complex skills studied in motor learning, racewalking offers advantages to better understand strategies of movement reorganization, first by allowing comparison to a reference initial pattern (i.e., walking gait) and second by the very nature of its biomechanical constraints that require a qualitative reorganization in movement coordinative patterns compared to normal walking (Murray et al. 1983; Majed et al. 2012; Pavei et al. 2014). The racewalking regulation imposes a straightened leg (i.e., no knee bending) from the moment of its first contact with the ground until its vertical upright position (IAAF 2016). Locomotor control has been extensively studied in the literature where ample data are available on normal and pathological walking patterns (Chau 2001; Kirkwood et al. 2011; Dillman et al. 2014). Many studies have described how the central nervous system controls the walking gait with only few independent components that seem to present consistent characteristics across subjects and different gait conditions such as walking at different speeds, stepping over an obstacle or walking with flexed knees (Lacquaniti et al. 2002; Ivanenko et al. 2007). These normal walking synergies were defined based on the fact that motions of the body’s limbs are controlled with respect to the direction of forward progression and that of gravity as the basic requirements for locomotion include dynamic equilibrium and postural stability (Borghese et al. 1996). From this perspective, it would be interesting to address the extent to which practicing a biomechanically constrained gait pattern influences normal walking synergies; thus adding to the available clinical research findings on the control of human gait.

In an earlier study examining movement reorganization with learning racewalking, Majed et al. (2012) found that practice-related kinematic changes occur early in practice (i.e., within four sessions). In this regard, the first aim of present study was to examine using PCA the extent to which the normal walking synergies (i.e., identified in a pretest) are preserved or modified over the course of four racewalk practice sessions. We expect to see invariant features in the gait patterns with practice, associated with a recruitment of a task-specific control dimension. The analysis will be done by evaluating common practice-related changes in (1) the number of relevant PCs and their highly loaded variables and (2) in the correlation coefficient of all studied variables.

## Methods

### Participants

Seven healthy participants with no previous experience in racewalking (2 females and 5 males, age 26.6 ± 1.8 years, weight 66.4 ± 7.5 kg, height 175.8 ± 6.9 cm, physical activity level 3.7 ± 1.5 h week^−1^) volunteered for this study. Prior to the experiment, participants signed a written informed consent in accordance with the Declaration of Helsinki. The experimental procedures were approved by the Ethical Committee of Paris-Sud University.

### Apparatus

A VICON motion analysis system connected to eight infrared emitting cameras (Oxford Metrics, UK) was used to record kinematic data at a sampling rate of 120 Hz. Nineteen reflective markers were placed at selected landmarks (plug-in-gait model set): xiphoid process, acromio-clavicular joints, lateral epicondyles of the elbow and knee, ulnar styloid processes, anterior superior iliac spines, exterior lateral lower 1/3 surface of the thighs, lateral malleoli, second metatarsals’ head and calcanei. All sessions were performed on a motorized treadmill (60 × 170 cm, 1–25 km h^−1^, Valiant, Lode, The Netherlands). Heart rate was monitored throughout all sessions using a Polar belt wrapped around the chest (Polar, Kempele, Finland).

### Protocol

The first laboratory visit consisted of a 30-min familiarization with the treadmill and warm-up followed by a standardized walk-to-run transition test to determine the individual preferred transition speed (PTS). In the next four visits, participants performed four racewalking practice sessions (S1–S4).

#### Preferred transition speed test

The protocol used to asses PTS is similar to that used in previous studies (Diedrich and Warren 1995; Majed et al. 2012). Participants were asked to perform four randomly given transition trials in which the treadmill speed was either incremented from 6 km h^−1^ (walk–run condition) or decremented from 10 km h^−1^ (run–walk condition) by steps of 0.5 km h^−1^ every minute. Treadmill velocity was kept constant between steps and participants, blind to the speeds, received the following instruction: “For these trials we will be changing the speed of the treadmill while you are on it. Please use the type of locomotion that feels most comfortable. That is, make the transition when it seems natural to do so.” The 1-min plateaus following the run-to-walk transition and corresponding to normal walking at PTS were analyzed and referred to as S0 (i.e., normal walking).

#### Racewalking practice sessions

The four practice sessions (S1–S4) were separated by at least 48 h and systematically started with a 10-min warm-up. Prior to each session, three instructions were given: (1) Foot contact with the ground should start with the heel, (2) advancing leg should remain straightened from the moment of its first contact with the ground until its vertical upright position (IAAF 2016) and (3) elbows should be flexed. In order to minimize discriminatory mechanical or physiological factors, the intensities used during practice were relative to the individual PTS (Hanna et al. 2000). The protocol’s structure, presented in Table 1, was inspired by a previous study (Majed et al. 2012), and the final learning goal was to racewalk for 4 min at PTS + 2 km h^−1^. All sessions comprised 4-min trials at PTS, PTS + 0.5, PTS + 1 and PTS + 1.5 km h^−1^ for comparison purposes. The overall practice duration was 28 min for S1 and 32 min for the subsequent sessions. Although higher intensities were gradually introduced, the overall practice duration did not exceed 32 min to ensure participants’ compliance. Indeed, the PTS + 1.5 and PTS + 2 km h^−1^ intensities were introduced in 2-min trials at S1 and S3, respectively, and were then increased in duration to 4-min trials at the following sessions (respectively). In order to allow participants to progressively reach the relative goal, the repetitions of trials at the lowest intensities (i.e., PTS and PTS + 0.5 km h^−1^) were decreased from two to one repetition at S3 and S4 as shown in Table 1. The rest periods between trials were monitored (HR < 120 bmp) using the heart rate data to ensure enough recovery time and avoid fatigue effects.

**Table 1:** Structure of practice sessions

Sessions’ structure: [number of trial’s repetition × trial duration (relative speed in km h^−1^)]	
S1	[2 × 4 min (PTS)] + [2 × 4 min (PTS + 0.5)] + [2 × 4 min (PTS + 1)] + [2 × 2 min (PTS + 1.5)]
S2	[2 × 4 min (PTS)] + [2 × 4 min (PTS + 0.5)] + [2 × 4 min (PTS + 1)] + [2 × 4 min (PTS + 1.5)]
S3	[1 × 4 min (PTS)] + [2 × 4 min (PTS + 0.5)] + [2 × 4 min (PTS + 1)] + [2 × 4 min (PTS + 1.5)] + [2 × 2 min (PTS + 2)]
S4	[1 × 4 min (PTS)] + [1 × 4 min (PTS + 0.5)] + [2 × 4 min (PTS + 1)] + [2 × 4 min (PTS + 1.5)] + [2 × 4 min (PTS + 2)]

### Data acquisition and analysis

In the walk-to-run transition test, the treadmill speed at which the gait transition occurred was determined by kinematic data (i.e., presence or absence of a flight phase). Individual PTS values were defined as the average of the four measured transition speeds (Hreljac 1995).

During practice sessions, kinematic data were collected in 30-s samples and recorded at the end of each 2-min trial (1:30–2:00 min). For the 4-min trials, an additional 30-s acquisition was carried out (3:30–4:00 min). Overall, out of 310 collected acquisitions, only two were discarded because of marker occlusions (i.e., marker fell or displaced during acquisition).

A custom MATLAB program (The MathWorks, USA) was used to compute movement variables (i.e., angular displacement) and run PCAs. Time series of eight key angular variables were computed from the original matrix of marker positions: angle of arm sagittal plane rotation, pelvis and thorax (shoulder line) frontal plane rotations, pelvis transverse plane rotation, elbow, hip, knee and ankle joint angles. Precisely, an overall of eight body segments were used for calculations (Majed et al. 2012, pp. 1603–1604). The shoulder, pelvis, forearm, arm, thigh, shank and foot segments represented, respectively, the lines connecting the markers of the left and right shoulders, left and right pelvis, elbow and wrist, elbow and shoulder, thigh and knee, knee and ankle and toe and heel. The trunk segment linked the sternum’s marker to the midpoint of the pelvis segment. Two types of angular displacements were computed (i.e., projected angles and joint angles). First, the shoulder and pelvis transverse rotations and the pelvis frontal rotation represented, respectively, the transverse and frontal projections of the angles between these segments and the laboratory medio-lateral axis. The arm sagittal rotation consisted of the sagittal projection of the angle formed with the laboratory vertical axis. Second, the hip, elbow, ankle and knee angles represented the absolute 3D angle between two segments, respectively; trunk and thigh, forearm and arm, foot and shank and shank and thigh. Time series were filtered with a second-order Butterworth low-pass filter (12 Hz cutoff frequency).

Prior to running PCA, stance phases of angle time series were retained for analysis and rescaled to unit variance so that variables with large amplitude do not influence the determination of PCs (Daffertshofer et al. 2004). For each practice trial, a PCA was performed resulting in computing 112 PCA on racewalking data (7 participants × 4 sessions × 4 speed trials) and 7 PCA on normal walking (7 participants). With the eigenvalues and eigenvectors obtained, the number of PCs required to capture a significant portion (i.e., 90%) of the total variance was assessed for each participant’s trial. PCs were sorted in a descending order of their respective amount of variance. The percentage of total variance accounted for by each of the first three PCs was also systematically analyzed. Variables’ loadings (weightings) onto each of the first three PCs were examined to have an insight on common trends explaining the role or nature of these PCs. The most representative movement variables within each PC were considered those with a relatively high loading value on a specific PC (i.e., eigenvector components higher than |0.4|, Stevens 2009). PCA was complemented with a correlation analysis of each of the normalized eight variables’ datasets for each trial in order to assess common patterns of changes in the degree of variables’ correlation.

### Statistical analysis

In order to assess immediate changes in gait patterns at the first practice trial (S1) compared to normal walking (S0) and after the instructions were given, all dependent variables (i.e., total variance explained by PC1, PC2 and PC3 and the number of PCs needed to capture 90% of total variance) were compared between S1 and S0 at PTS using Wilcoxon’s tests with respect to the normality of data sets verified by Shapiro–Wilk’s test. The effect size values were described by the magnitude of change expressed as Cliff’s delta (|*r*|) to give a rigorous judgment about the differences between S0 and S1.

The effect of practice on gait patterns was examined by comparing variables within the four sessions at the common relative speeds (i.e., PTS, PTS + 0.5, PTS + 1 and PTS + 1.5 km h^−1^) using Friedman tests (normality tested using Shapiro–Wilk’s procedure). Post hoc pairwise comparisons were done when needed using Wilcoxon’s tests with a Bonferroni adjustment.

A correlation analysis examined the correlation coefficient between each of the eight individual normalized variables’ datasets for each participant. Using Wilcoxon’s tests, the mean correlation coefficients (Spearman’s rho) were then compared between S0 and S1 at PTS and between early practice (S1) and late practice (S4) at the four common relative speeds. This analysis was done to identify common strategies in the control of movement reorganization.

All tests were performed with Statistica 7.1 package (Statsoft 2005) with a level of significance set at *p* < 0.05.

## Results

### Normal walking at PTS (S0)

**Fig. 1:**
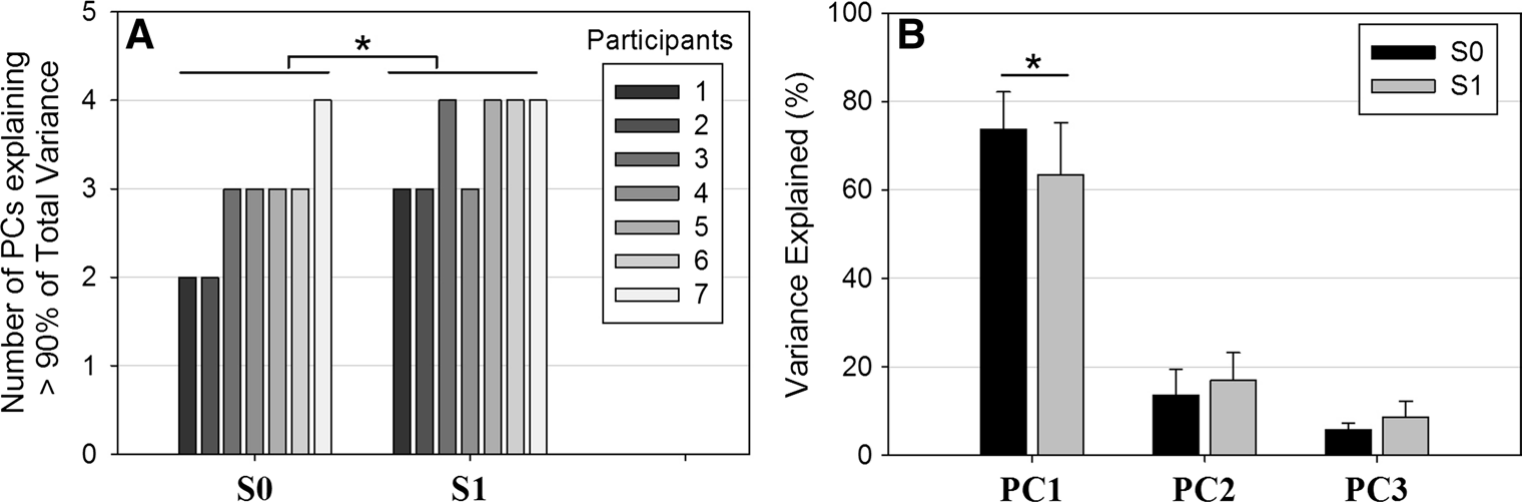
**a** Number of PCs explaining more than 90% of total variance for S0 and S1 (PTS). Data of all participants (# 1–7) are presented for better visualization. **b** Mean percentage of total variance explained by each of the first three PCs for S0 and S1 (PTS). *Error bars* represent within-participant standard deviation. *p < 0.05

On average, participants exhibited a PTS of 7.29 ± 0.70 km h^−1^, indicating the speed above which walking was no longer the preferred pattern compared to running. Results showed that at S0, the number of relevant PCs varied between two and three for most participants (expect for one participant that needed four PCs to explain 90% of total variance) with a median value of three PCs (Fig. 1a). Table 2 shows the general trends in the distribution of highly loaded variables within each PC. For the majority of participants at S0, it is clear that PC1 included motions of the arm (without exceptions), hip, shoulder (transverse plane), pelvis (transverse plane) and elbow, and PC2 encompassed the knee motion and pelvis frontal rotation while PC3 did not reveal common features between the majority of participants (Table 2).

**Table 2:**
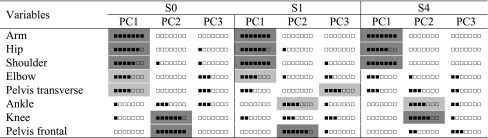
Common trends in the distribution of highly loaded variables within the first three PCs for the participants’ PTS trials for normal walking (S0) and first (S1) and last sessions of practice (S4)

For each variable within each PC, black square stands for a trial where the corresponding variable was highly loaded and white square for a trial where the variable’s loading was low. For a better visualization, color boxes indicate that a variable was highly loaded for more than 5 out of 7 participants (dark gray) or for 4 out of 7 participants (light gray)

### First racewalk trial at S1 and immediate changes compared to normal walking (S0)

Table 2 shows that at S1 the majority of participants had similar trends in the organization of highly loaded variables within PC1 compared to S0. Indeed, the motions of the arm (for all participants), hip, shoulder and elbow were still highly loaded onto PC1, while PC2 clearly encompassed the motion of the pelvis frontal rotation. Although no common features were found between participants for PC3 at S1, a majority of them had the transverse pelvis motion contributing to the variance of PC3.

At the first practice trial, results showed that the number of relevant PCs varied between three and four with a median value of four PCs (Fig. 1a). The Wilcoxon’s test indicated that significantly more PCs were needed to account for 90% of total variance at S1 compared to S0 [*Z* = 2.023, |*r*| = 0.551, *p* < 0.05] (Fig. 1a). Given that some participants did not need four PCs to account for the majority of total variance at S1 but only three PCs, we chose here to systematically examine changes in the variance of the first three PCs. As indicated in Fig. 1b, the percentage of total variance explained by PC1 decreased significantly from 73.70 ± 8.44% at S0 to 63.40 ± 11.76% at S1 [*Z* = 2.367, |*r*| = 0.551, *p* < 0.05], whereas the variance of PC2 and PC3 did not change significantly between S0 and S1.

**Fig. 2:**
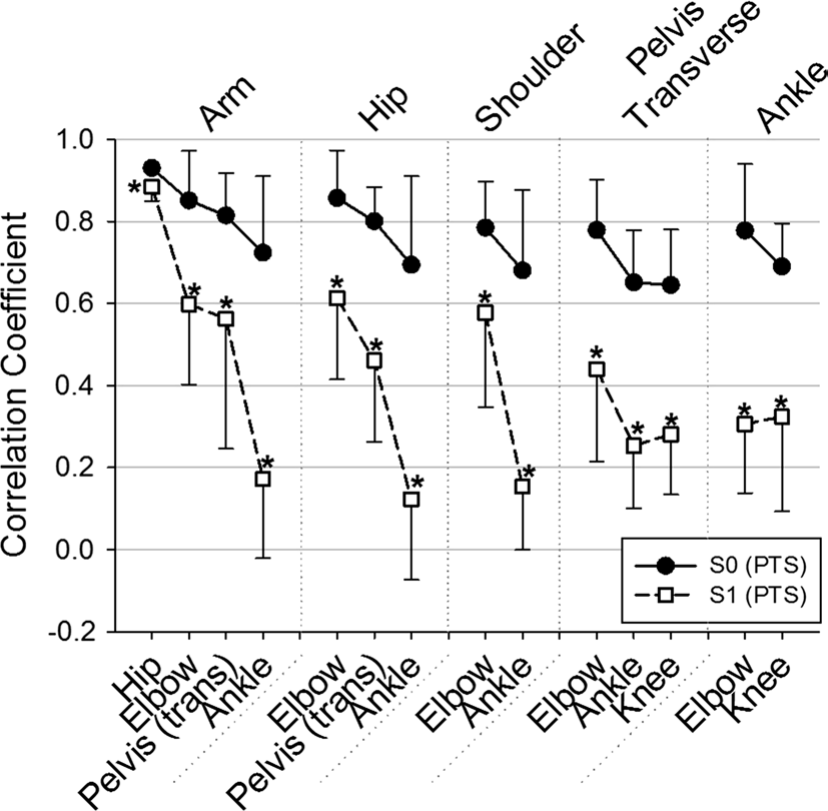
Mean correlation coefficient values for S0 and S1 at PTS between each of the variables presented on the *x*-axis and the motion of the arm, hip, shoulder, pevis (transverse plane) and ankle angles. Only correlations that presented a significant change between S0 and S1 are presented here. *Error bars* represent within-participant standard deviation. *p < 0.05

The comparison of the Spearman’s correlation coefficients between S0 and S1 (all pairwise correlation were significant at all trials, *p* < 0.05) showed a general decrease in the correlation coefficient between several body parts at S1 compared to S0, while no increases in the correlation between variables were noted. Specifically, Fig. 2 depicts the significant reduction in the degree of correlation within variables highly loaded onto PC1 and between the latter and the pelvis (transverse), knee and ankle motions (*p* < 0.05), respectively. These results support the general trends found in Table 2. Namely, significantly less correlation was found at S1 compared to S0 between the motions of the arm and hip [*Z* = 2.366, |*r*| = 0.918], elbow [*Z* = 2.366, |*r*| = 0.796], pelvis (transverse) [*Z* = 2.366, |*r*| = 0.551] and ankle [*Z* = 2.366, |*r*| = 0.918], respectively; between the motions of the hip and elbow [*Z* = 2.366, |*r*| = 0.878], pelvis (transverse) [*Z* = 2.366, |*r*| = 0.959] and ankle [*Z* = 2.366, |*r*| = 0.878], respectively, and between the motions of the shoulder and elbow [*Z* = 2.028, |*r*| = 0.673] and ankle [*Z* = 2.366, |*r*| = 0.918], respectively. A significant decrease in the correlation coefficient at S1 compared to S0 was also found between the motions of the pelvis (transverse) and elbow [*Z* = 2.197, |*r*| = 0.918], ankle [*Z* = 2.366, |*r*| = 1.000] and knee [*Z* = 2.366, |*r*| = 0.918], respectively, and between the motions of the ankle and elbow [*Z* = 2.366, |*r*| = 0.918] and knee [*Z* = 2.366, |*r*| = 0.918], respectively.

### Practice-related changes

**Fig. 3:**
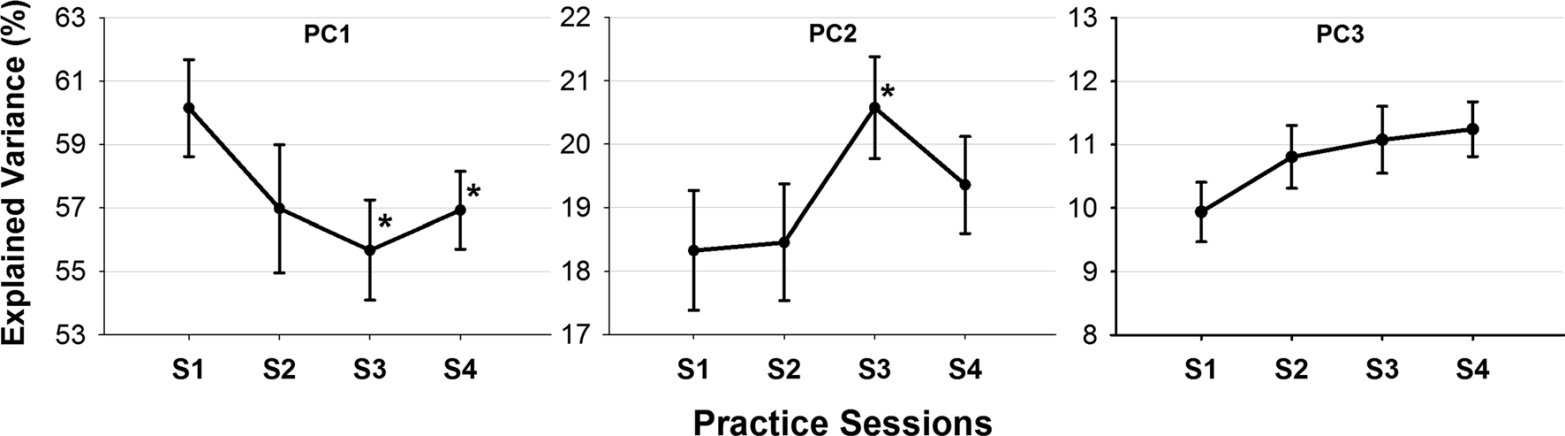
Mean percentage of the total variance explained by each of the first three PCs as a function of practice sessions. *Error bars* represent the standard error. *Significantly different compared to S1 (*p* < 0.05)

Table 2 shows that at S4, PC1 still clearly encompassed the motions of the arm, hip and shoulder as observed at S0 and S1. Compared to normal walking and early practice, PC2 also showed high loadings for the motions of the ankle and knee (lower extremities), while no common trends were evident for PC3. The Friedman comparison indicated no significant practice-related changes in the number of PCs required to capture 90% of total variance [median values of “four PCs” at S1 and S4, *χ* ^2^(3) = 3.632, *p* = 0.30]. However, considering the variance explained by each PC, results indicated a significant decrease for PC1 [*χ* ^2^(3) = 10.249, *p* = 0.0126] and a significant increase for PC2 [*χ* ^2^(3) = 10.586, *p* = 0.014], while no changes were noted for PC3 [*χ* ^2^(3) = 6.471, *p* = 0.09]. As shown in Fig. 3, post hoc analysis revealed that the significant changes in the variance explained by PC1 and PC2 happened between S1 and S3 [*Z* = 2.505, |*r*| = 0.298, *p* < 0.05; *Z* = 2.482, |*r*| = 0.347, *p* < 0.05, respectively] and between S1 and S4 [*Z* = 2.869, |*r*| = 0.436, *p* < 0.01] only for PC1.

**Fig. 4:**
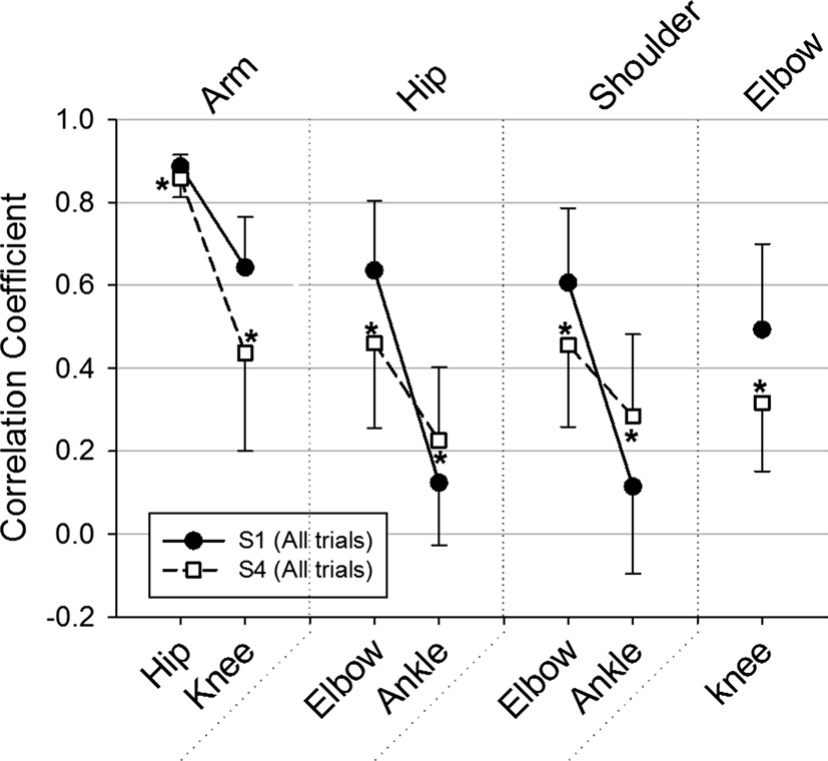
Mean correlation coefficient values for S1 and S4 at all relative speed trials between each of the variables presented on the *x*-axis and the arm, hip, shoulder and elbow angles. Only correlations that presented a significant change between S1 and S4 are presented here. *Error bars* represent within-participant standard deviation. *p < 0.05

The significant practice-related changes in the correlation coefficients are presented in Fig. 4. Results showed a further significant decrease in the correlations within variables highly loaded onto PC1 and between the latter and the knee motion. Namely, those differences were seen between the motions of the arm and hip [*Z* = 2.366, |*r*| = 0.469] and knee [*Z* = 0.197, |*r*| = 0.510], respectively, and between the motions of the hip and elbow [*Z* = 2.366, |*r*| = 0.469], shoulder and elbow [*Z* = 2.366, |*r*| = 0.469] and elbow and knee [*Z* = 2.028, |*r*| = 0.551]. However, significant increases in the correlation coefficients between the motions of the ankle and hip [*Z* = 2.366, |*r*| = 0.755, *p* < 0.05] and shoulder [*Z* = 2.028, |*r*| = 0.510, *p* < 0.05], respectively, were also noted (Fig. 4).

## Discussion

This study challenged to identify common control strategies in movement reorganization when learning the biomechanical constraints of a differentiated gait pattern (i.e., racewalking) by using PCA. Main findings of the present study indicated that: (1) Although common features of normal gait were preserved over the course of practice, more PCs were needed to explain the data’s variance directly at S1 compared to normal walking (S0), (2) the variance explained by PC1 decreased immediately at S1 (compared to S0) and further with practice, while the variance explained by PC2 increased significantly with practice. Although a decrease in the correlation of many variables was evident at S1 (compared to S0) and with practice, the ankle’s motion increased its correlation with variables highly loaded on PC1.

### At the initial normal walking pattern (S0)

The PC analysis extracted two to four distinct control dimensions for all participants, with a median value of three PCs (Fig. 1a). For the initial walking gait, the first PC accounted for the largest portion of movement’s variance (i.e., 73.7%) for all participants, while values for PC2 and PC3 were 13.55 and 5.74%, respectively. The first PC was fairly robust and presented common trends across all participants encompassing mainly the motions of the arm and also hip and transverse shoulder rotation (Table 2). PC2 was also consistent between participants as it correlated mainly with the knee motion and the frontal pelvis rotation while no common features were identified for PC3 at S0. A possible interpretation of the role of these two main components is that PC1 reflected an upper-extremities/antero-posterior control dimension (i.e., progression) while PC2 explained better a lower-extremities/vertical control dimension (i.e., body sway or shock absorption at the stance phase). This interpretation corroborates previous kinematic findings indicating that human walking can be reduced to two independent components. For instance, although variables’ waveforms were not examined, it is interesting to note that Daffertshofer et al. (2004) suggested an independent control by the CNS of variables that oscillate at the same frequency as the stride frequency (i.e., PC1 in the present study) and those that oscillate at twice the basic frequency (i.e., PC2 in the present study). In addition, Ivanenko and Lacquaniti’s work (Lacquaniti et al. 2002; Ivanenko et al. 2007) suggested that the CNS may operate by controlling limb endpoint in walking with two separate components linked first to the limb orientation or stride length (i.e., PC1 in the present study) and second to the limb’s length or loading (i.e., PC2 in the present study). Although, their studies focused mainly on synergies of lower limbs, the control mechanism could be generalized to upper limb control in walking. Given the robustness of PC1 that clearly and systematically encompassed the arm motion, another noteworthy interpretation, originating from studies on dynamic postural control during quadrupedal and bipedal gaits, suggests the importance of upper extremity control in gait (Earhart 2013). Indeed, strong evidence from studies on healthy and impaired gait reveals clear links between locomotor control and upper extremity control (Nieuwboer et al. 2009; Vercruysse et al. 2012; Earhart 2013) which seem to support our findings. In this framework, a question arises concerning the extent to which these “normal synergies” of walking gait are maintained after practicing an artificial walking pattern that places specific biomechanical demands on the loading component (i.e., PC2 in the present study) by constraining the bending of the knee at stance.

### At the first trial of racewalk practice (S1)

After the instructions were given, PC analysis extracted three to four distinct control dimensions for all participants. Compared to normal walking (S0), a significant increase in the number of PCs was needed to capture a significant portion of movement variance (i.e., 90%) at S1. According to Hong and Newell (2006), an increase in the number of retained components could be interpreted as a “recruitment” of an additional control dimension, seen here in five out of seven participants (Fig. 1a). At this stage, the interpretation of a “recruitment” strategy is hard to confirm given that a closer look at the PCs’ characteristics indicated a significant decrease in the variance accounted for by PC1 while no increases in subsequent PCs were statistically significant at S1 compared to S0 (Fig. 1b). Even though PC1 still accounted for the largest portion of movement variance (i.e., 63.39%) at S1 and presented common trends across participants, it is possible that this first “upper-extremities/antero-posterior” component was weakened. This was supported by the general significant reduction at S1 (compared to S0) in the strength of correlations between variables highly loaded on PC1, which were also significantly less correlated with motions of the knee, ankle and pelvis transverse rotation (Fig. 2). This could be seen as a strategy of destabilizing spontaneous tendencies (i.e., PC1) to facilitate the adoption of more individuated task-specific coordination modes (Furuya et al. 2014). These interpretations go in line with results of Majed et al. (2012) that found an immediate general increase in the variability of similar motions (i.e., hip, elbow, pelvis transverse rotation) when practicing the racewalking’s constraints (compared to S0). It is worth noting that even though PC2 still encompassed “lower-extremities/vertical motions” (i.e., frontal pelvis motion, ankle) at S1, more participants dissociated the knee motion from this second component at S1 (compared to S0). This was confirmed by the significant reduction observed in the degree of correlation between the knee and ankle motion (Fig. 2). Given the constraint imposed on the knee (i.e., no bending during stance phase), it is logical to assume an effort done by most participants at S1 to dissociate its motion from those believed to present a double oscillation per stride (i.e., PC2). Finally, results also indicated that more participants at S1 (compared to S0) dissociated the pelvis transverse rotation from variables highly loaded on PC1 (Fig. 2) and shifted its relative contribution to a third independent control dimension (i.e., PC3) (Table 2). This could better support the idea of a “recruitment” that is not directly linked to a need of an additional control dimension per se, but rather to a shift in the relative contribution of some task-specific variables onto different PCs. In a sense, the robustness of the first two PCs is revealed by their consistent features across participants; however, the control of the reorganization in movement is rather seen by a dissociation of some task-specific variables from these “normal synergies.”

### With advancement in the practice sessions

While no additional PCs were retained as compared to early practice, results indicated a significant [further] decrease in the variance accounted for by PC1 and a significant increase in the contribution of subsequent PC2 to total variance (Fig. 3). This was associated with a [further] weakening with practice of the strength of the correlations between variables initially highly loaded on PC1 (i.e., arm–hip, hip–elbow, shoulder–elbow) and between the latter and the knee (i.e., arm–knee) (Fig. 4). Hong and Newell (2006) have proposed that any increase with practice in the variance accounted for by a component is suggestive of an increasing stability of the various coordination modes. Considering this interpretation, our results could suggest a reduction with practice in the stability of the first upper-extremities/antero-posterior component and an increase in that of the lower-extremities/vertical component (i.e., PC2) that seemed to play a more specific role in maintaining the task-related constraints as it clearly (re-)encompassed the motions of the knee and ankle at S4. It is important to note that the analysis performed for the practice sessions included speeds above the range at which walking is freely chosen. This could have further reduced the dynamic stability, specifically in the forward progression direction, which further corroborates the interpretation of a destabilization in PC1. PC2 seemed rather reinforced with practice and characterized by a further dissociation of the knee from variables highly loaded on PC1. Although PC1 and PC2 still presented similar common features across participants at S4, PC3 did not present any shared features across participants at S4. According to the motor learning literature, a suppression of control dimensions (i.e., here PCs) could be expected with practice (Chen et al. 2005; Verrel et al. 2013). However, the results of this study do not allow us to support the “suppression” hypothesis. We believe that one of the limitations of this study is the lack of understanding of a clear role of PC3 for the majority of participants that could be explained by the adoption of individualized strategies that are not captured by our analysis or by possible shifts between PC3 and PC4 (not examined here) across participants and/or trials. A further study would be needed to address this limitation that does not affect our main findings on the roles and changes in PC1 and PC2. Finally, we believe that even if PC1 and PC2 were destabilized or reinforced with practice, our results go in line with the existence of invariant predictors for the control of walking gait as found by Ivanenko et al. (2007) for limb control during normal walking and for different gait conditions.

### In sum

PCA were used to evaluate changes in the control strategies occurring in the learning of a biomechanically constrained walking pattern, racewalking. Before practice, normal walking was characterized by two predominant PCs explaining, respectively, the upper-extremities/antero-posterior (i.e., progression) and the lower-extremities/vertical (i.e., loading) motions. Compared to normal walking, the immediate increase at S1 in the number of PCs required to capture a significant portion of movement variance could be suggestive of an immediate “recruitment” of an additional control dimension to fulfill the task-specific constraints. On one hand, although common features (i.e., synergies) for the two first PCs were preserved with practice, the decrease in variance of PC1 could indicate a destabilization of spontaneous tendencies of the upper-extremities/antero-posterior control component to facilitate the adoption of more task-specific coordination modes. This interpretation is further justified by the general decrease with practice in the strength of the correlations between highly loaded variables on PC1. On the other hand, the increase in the variance accounted for by PC2 suggests a reinforcement of this lower-extremities/vertical component that could reflect efforts to fulfill the constraints placed on the ankle and knee (blocking). To our knowledge, this study is the first that used PCA to extract common practice-related changes in the control of whole-body action (i.e., upper and lower limbs and trunk motions), and to extend on invariant control characteristics of the normal walking gait that could be considered for clinical assessments and future research studies.
